# Self-Organization in Dilute Aqueous Solutions of Thermoresponsive Star-Shaped Six-Arm Poly-2-Alkyl-2-Oxazines and Poly-2-Alkyl-2-Oxazolines

**DOI:** 10.3390/polym13091429

**Published:** 2021-04-29

**Authors:** Tatyana Kirila, Anna Smirnova, Vladimir Aseyev, Andrey Tenkovtsev, Heikki Tenhu, Alexander Filippov

**Affiliations:** 1 Institute of Macromolecular Compounds of the Russian Academy of Sciences, Bolshoy pr., 31, 199004 Saint Petersburg, Russia; av.smirnova536@gmail.com (A.S.); avt@hq.macro.ru (A.T.); afil@imc.macro.ru (A.F.); 2 Department of Chemistry, University of Helsinki, 00014 Helsinki, Finland; vladimir.aseyev@helsinki.fi (V.A.); heikki.tenhu@helsinki.fi (H.T.)

**Keywords:** thermoresponsive star-shaped polymers, poly-2-alkyl-2-oxazines and poly-2-alkyl-2-oxazolines, aqueous solutions, light scattering, turbidimetry, microcalorimetry, phase separation, aggregation

## Abstract

The behavior of star-shaped six-arm poly-2-alkyl-2-oxazines and poly-2-alkyl-2-oxazolines in aqueous solutions on heating was studied by light scattering, turbidimetry and microcalorimetry. The core of stars was hexaaza [2_6_] orthoparacyclophane and the arms were poly-2-ethyl-2-oxazine, poly-2-isopropyl-2-oxazine, poly-2-ethyl-2-oxazoline, and poly-2-isopropyl-2-oxazoline. The arm structure affects the properties of polymers already at low temperatures. Molecules and aggregates were present in solutions of poly-2-alkyl-2-oxazines, while aggregates of two types were observed in the case of poly-2-alkyl-2-oxazolines. On heating below the phase separation temperature, the characteristics of the investigated solutions did not depend practically on temperature. An increase in the dehydration degree of poly-2-alkyl-2-oxazines and poly-2-alkyl-2-oxazolines led to the formation of intermolecular hydrogen bonds, and aggregation was the dominant process near the phase separation temperature. It was shown that the characteristics of the phase transition in solutions of the studied polymer stars are determined primarily by the arm structure, while the influence of the molar mass is not so significant. In comparison with literature data, the role of the hydrophobic core structure in the formation of the properties of star-shaped polymers was analyzed.

## 1. Introduction

Thermoresponsive pseudo-polypeptoids have attracted great interest of researchers in recent years due to the wide potential of their application, for example, in medicine as nanocontainers for targeted delivery of drugs [1,2,3]. For them, the synthesis conditions were determined, allowing obtaining polymers with a given structure and molar mass characteristics. The linear pseudo-polypeptoids have been studied in sufficient detail, and influence of their chemical structure and molar mass on the physico-chemical properties, in particular, on self-organization of macromolecules and thermoresponsiveness in aqueous solutions was established [4,5,6,7,8,9,10].

One of the most well-studied classes of thermosensitive pseudo-polypeptoids is poly-2-alkyl-2-oxazolines (PAlOx). They demonstrate LSCT behavior in water-salt solutions, and the phase separation temperature depends on the length of the side radical and can range from practically zero to 100 °C [11]. Due to their biocompatibility and stability in biological media, they are widely used in medical applications and biotechnology [2,12,13]. In particular, complexes of linear PAlOx with low molecular weight compounds were obtained, which are used as delivery systems for drugs, DNA, as well as materials for creating biocompatible composite structures [14].

Poly-2-alkyl-2-oxazines (PAlOz) are homologs of poly-2-alkyl-2-oxazolines. The presence of an additional methylene group in the monomer unit makes them more hydrophobic than PAlOx [15,16,17]. In contrast to poly-2-alkyl-2-oxazines, PAlOz have been studied in much less detail [18], although the synthesis of these polymers was described at about the same time [19]. This situation is associated with the difficulties of synthesis of PAlOz, namely, the reaction for the preparation of PAlOz is characterized by low polymerization rate constants and a high chain transfer rate. This makes it difficult to obtain the high molar mass samples [20,21]. It should be noted that PAlOz has a number of advantages over PAlOx. For PAlOz, no irreversible crystallization in water was found upon prolonged heating above the phase separation temperature, which is characteristic of poly-2-isopropyl-2-oxazoline [22,23]. It seems even more significant that for PAlOz good binding of water-insoluble medicinal compounds was found [24].

In connection with biomedical applications, special attention is paid to the study of pseudo-polypeptoids with complex architecture, in particular, polymer stars. For star-shaped PAlOx, the effect of the structure of arms, their number and length, as well as the solvent composition on the thermosensitivity was analyzed [25,26,27]. The possibilities of using star-shaped polymers as carriers of drugs to a great extent depend on the structure and properties of the branching center. For example, calix[*n*]arenes and resorcinarenes are prone to self-organization and the formation of nanoscale assemblies [28,29]; therefore, their use as the core of star molecules significantly increases the binding efficiency of low-molecular-weight drugs.

Star-shaped polymers based on aza [1_n_] cyclophans have practically not been described so far, although the aza [1_n_] cyclophans have been known of since 1963 [30]. Hexaase [2_6_] metacyclophane and hexaase [2_6_] orthoparacyclophane can be obtained in high yield by reduction in the corresponding macrocyclic Schiff bases [31].

**Figure 1:**
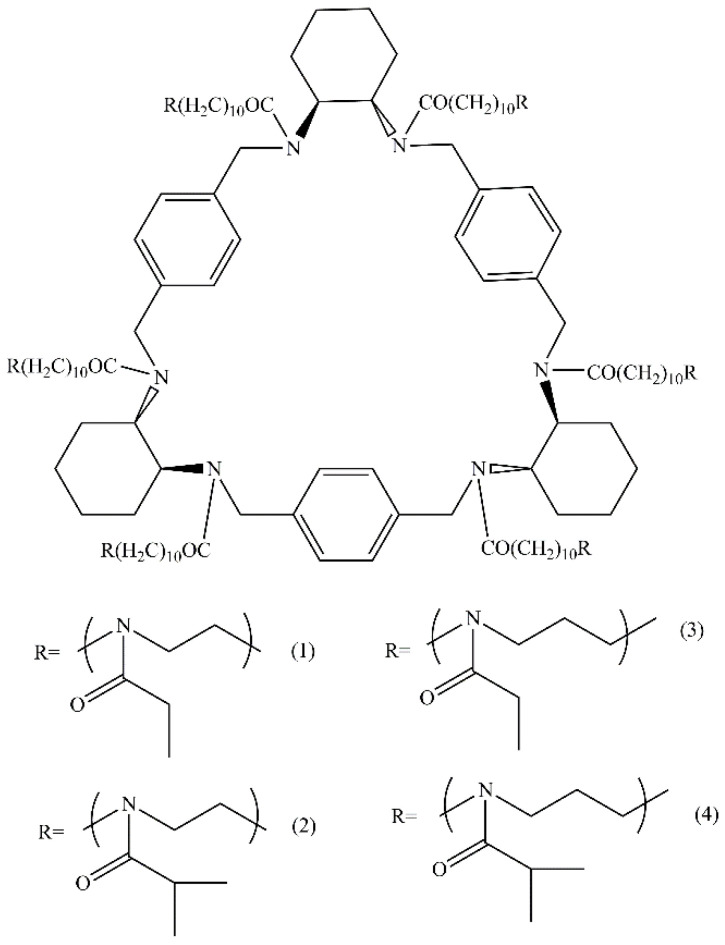
Structure of star-shaped CPh6-PEtOx (**1**), CPh6-PiPrOx (**2**), CPh6-PEtOz (**3**), and CPh6-PiPrOz (**4**).

In our previous work [25], for the first time, star-shaped six-arm pseudo-polypeptoids with hexaaza [2_6_] orthoparacyclophane core were synthesized using cationic ring-opening polymerization. Four polymers were obtained, namely, star-shaped poly-2-ethyl-2-oxazine (CPh6-PEtOz), poly-2-isopropyl-2-oxazine (CPh6-PiPrOz), poly-2-ethyl-2-oxazoline (CPh6-PEtOx), and poly-2-isopropyl-2-oxazoline (CPh6-PiPrOx) (Figure 1). Conformational behavior of star-shaped poly-2-alkyl-2-oxazines (CPh6-PAlOz) and poly-2-alkyl-2-oxazolines (CPh6-PAlOx) were investigated by the methods of molecular hydrodynamics and optics in molecular dispersed solutions. It was established that conformation characteristics of CPh6-PAlOz and CPh6-PAlOx depended on arm length, while the chemical structure weakly affected the solution behavior of the star-shaped pseudo-polypeptoids. The star-shaped CPh6-PAlOz and CPh6-PAlOx are characterized by higher intramolecular density in comparison with their linear analogs. Taking into account the prospects of practical application, the influence of salt on the self-organization in CPh6-PAlOz and CPh6-PAlOx solutions was studied [32]. NaCl and N-methylpyridinium p-toluenesulfonate (*N*-PTS) were used as salts. It was found that the effect of salt on the thermosensitivity of the discussed stars depends on the structure of the salt and polymer and on the salt content in the solution. For NaCl solutions, the phase separation temperature monotonically decreased with the growth of salt concentration. In *N*-PTS solutions, the dependence of the phase separation temperature on the salt concentration was non-monotonic with minimum at salt concentration corresponding to one salt molecule per one arm of a polymer star.

The aim of the present work is to investigate the effect of the arm structure and, accordingly, the hydrophobicity of the molecules on self-organization and aggregation in water solutions of thermoresponsive star-shaped CPh6-PAlOz and CPh6-PAlOx on heating.

## 2. Materials and Methods

### 2.1. Polymer Star Synthesis

The synthesis of star-shaped six-arm thermosensitive poly-2-alkyl-2-oxazolines and poly-2-alkyl-2-oxazines with a hexaase [2_6_] orthoparacyclophane core has been described in detail earlier [25]. The molar mass characteristics of the samples were determined in chloroform. The molar masses (MM) of CPh6-PEtOz and CPh6-PiPrOz are higher than MM of CPh6-PEtOx and CPh6-PiPrOx (Table 1). Accordingly, the molar fraction *ω* of hydrophobic groups in CPh6-PEtOz (7.2 mol %) and CPh6-PiPrOz (8.3 mol %) is almost one and a half times lower than in CPh6-PEtOx (11.0 mol %) and CPh6-PiPrOx (11.8 mol %). The values of dispersity factor *Đ* = *M*_w_/*M*_n_ of investigated samples were calculated using GPC curves which were obtained in [25]. However, it should be taken into account that linear standards were used in the GPC analysis. Accordingly, the obtained *Đ* values should be considered as an estimate of the polydispersion.

### 2.2. Solution Investigation

The thermosensitive behavior of star-shaped pseudo-polypeptoids was studied by light scattering and turbidimetry on a PhotoCor Complex (Photocor Instruments Inc., Moscow, Russia) setup with a diode laser and a sensor for measuring optical transmission. Wavelength is *λ* = 659.1 nm. The studies were carried out in the temperature range *T* from 5 to 75 °C. The value of *T* was changed discretely with a step from 0.5 to 5 °C and was regulated with an accuracy of 0.1 °C.

The measurement procedure was described in detail [33]. For all solutions, the values of the scattered light intensity *I*, optical transmission I*, hydrodynamic radii *R*_h_ of scattering objects and their *S_i_* contribution to the total light scattering were determined. The *S*_i_ values were obtained from the area of the corresponding peak in the intensity distribution *I* over *R*_h_.

**Figure 2:**
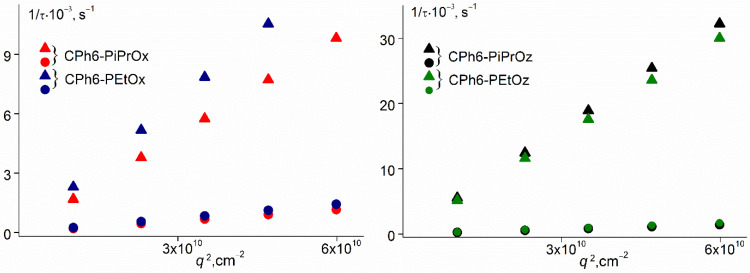
Relaxation time 1/τ on squared wave vector *q*^2^ for CPh6-PAlOz and CPh6-PAlOx at 21 °C.

After the given temperature was established, only the dependences of the light scattering intensity *I* and optical transmission I* on time *t* were recorded. These measurements were carried out at a scattering angle of 90°. When the changes in *I* were no more than 1% over the time required for the accumulation and processing of the autocorrelation function, the distribution of *I* over the hydrodynamic radii *R*_h_ was obtained, and the angular dependences of the values of *I*, *R*_h_, and *S*_i_ were analyzed in the range of light scattering angles from 45° to 135° in order to prove the diffusion nature of the modes, as well as to obtain extrapolated values of *R*_h_ (Figure 2). In all experiments, the time *t*_eq_ required to achieve time constant values of *I*, *R*_h_, and *S*_i_ was recorded. Since the phase separation temperatures for CPh6-PEtOx and CPh6-PEtOz at *c* < 0.01 g cm^−3^ had values above 75 °C, measurements for these solutions were carried out on a Zetasizer Nano ZS (Malvern Instruments Limited, Worcestershire, UK) particle analyzer in the temperature range from 15 to 100 °C.

The polymer concentrations varied from 0.0002 to 0.015 g cm^−3^ for CPh6-PiPrOx and CPh6-PiPrOz and from 0.005 to 0.025 g⋅cm^−3^ for CPh6-PEtOx and CPh6-PEtOz. The solutions were filtered into dust-free vials using Millipore filters with hydrophilic PTFE membrane and pore size of 0.45 µm (Merck KGaA, Darmstadt, Germany).

Microcalorimetric studies of aqueous solutions of star-shaped CPh6-PAlOz and CPh6-PAlOx were carried out on a MicroCal PEAQ-DSC microcalorimeter (Malvern Instruments Limited, Worcestershire, UK) with a capillary cell with a volume of 0.507 mL at a heating rate of 1 °C/min and a pressure of 50 kPa. The temperature range of measurements was chosen depending on the characteristics of the polymer (from 5 to 60 °C for CPh6-PiPrOz and from 15 to 100 °C for CPh6-PiPrOx, CPh6-PEtOx, and CPh6-PEtOz). The solution concentrations varied from 0.001 to 0.015 g cm^−3^ for CPh6-PiPrOx and CPh6-PiPrOz, and from 0.005 to 0.025 g cm^−3^ for CPh6-PEtOx and CPh6-PEtOz. The phase transition heat Δ*H* was calculated using the OriginLab taking into account the polymer concentration.

## 3. Results and Discussion

### 3.1. Behavior of Star-Shaped Six-Arm Pseudo-Polypeptoids in Aqueous Solutions at Low Temperatures

**Figure 3:**
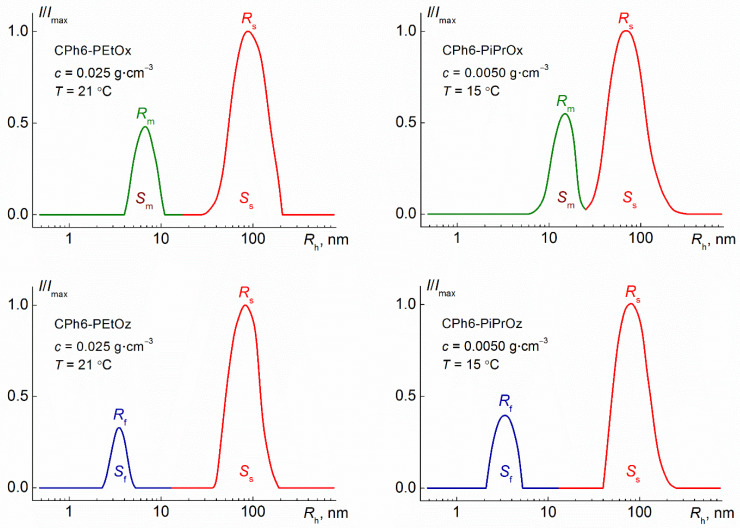
The distributions of light scattering intensity over hydrodynamic radii of scattering species for solutions of star-shaped pseudo-polypeptoids. *R*_f_, *R*_m_, and *R*s, are the hydrodynamic radii of macromolecules (fast mode), small aggregates (middle mode) and large aggregates (slow mode), respectively. *S*_f_, *S*_m_, and *S*_s_ are contributions of the corresponding modes to the total light scattering.

**Figure 4:**
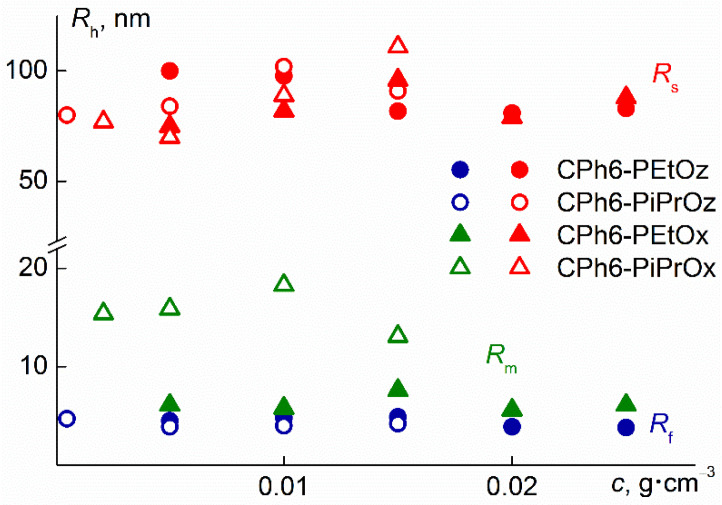
The dependences of the hydrodynamic radii *R*_h_ of scattering species on concentration *c* for aqueous solutions of star-shaped CPh6-PEtOz and CPh6-PEtOx 15 °C and CPh6-PiPrOz and CPh6-PiPrOx at 21 °C.

At low temperatures, the aqueous solutions of the studied polymer stars were not molecularly dispersed; there were two types of scattering objects at all concentrations (Figure 3). However, the behavior of CPh6-PAlOz and CPh6-PAlOx was markedly different. In the case of CPh6-PEtOz and CPh6-PiPrOz, the radii *R*_f_ of the particles responsible for the fast mode (Table 1) were close to the hydrodynamic radii *R*_h-D_ of the macromolecules of the stars under consideration, determined in an organic solvent in which there were no associative phenomena [25]. Therefore, particles with radius *R*_f_ could be considered as individual star-shaped macromolecules. Taking into account the hydrophobicity of the CPh6 core and the hydrophilicity of the PEtOz arms, it can be assumed that the structure of CPh6-PAlOz molecules in aqueous solutions is similar to that of unimolecular micelles of the core-shell type [34,35,36,37]. For both PAlOz stars, the *R*_f_ values did not depend on the polymer concentration (Figure 4).

In exactly the same way, no concentration dependence was found for the species responsible for the faster mode in CPh6-PEtOx and CPh6-PiPrOx solutions. However, the radii *R*_m_ of these particles differed markedly from *R*_h-D_ for CPh6-PAlOx. For CPh6-PEtOx, the concentration-averaged value <*R*_m_> = (6.3 ± 0.7) nm is 2.1 times higher than *R*_h-D_ = 3.0 nm. For CPh6-PiPrOx, the discussed parameters differed more strongly: <*R*_m_> = (15 ± 1) nm and *R*_h-D_ = 2.6 nm. Therefore, the species under discussion are supramolecular structures. Similar aggregates, which are usually called micelle-like structures, have been repeatedly observed for stimulus-sensitive polymers [6,35,38,39,40,41,42], including the star-shaped PAlOx and PAlOz [43,44,45,46]. The formation of these supramolecular structures in solutions of the studied CPh6-PAlOx is mainly caused by interaction of hydrophobic CPh6 core. This explains the difference in the behavior of CPh6-PAlOx and CPh6-PAlOz. In the CPh6-PEtOz and CPh6-PiPrOz molecules, the hexaaza [2_6_] orthoparacyclophane core is well shielded from water molecules by the hydrophilic corona formed by the arms, that prevents the formation of aggregates. The lengths *L*_tsc_ of the chains of PEtOz and PiPrOz are 15.9 and 12.1 nm [25]. These values are almost two times higher than *L*_tsc_ for CPh6-PEtOx (*L*_tsc_ = 8.5 nm) and CPh6-PiPrOx (*L*_tsc_ = 6.9 nm). Accordingly, in the case of CPh6-PAlOx, the arm length is not enough for reliable screening of branching centers, and the interaction of the latter leads to the aggregate formation.

As was shown earlier, the shape of micelle-like aggregates is close to spherical [45]. The spherical form of molecules of polymer stars with a large number of relatively short arms was proved theoretically [47] and experimentally [48]. These facts allow the estimation of the aggregation degree *m*_agg_ as the ratio of volumes of the aggregates and the macromolecules. For spherical particles, the volume is proportional to third power of their radius and for investigated CPh6-PAlOx

*m*_agg_ ≈ (*R*_m_/*R*_h-D_)^3^ (1)

Thus, *m*_agg_ ≈ 9 and 200 for CPh6-PEtOx and CPh6-PiPrOx, respectively. Note that such an approach is rough. First, the hydrodynamic radius is not the real dimension of dissolved species. Second, the densities of aggregates and macromolecules are different. A significant distinction in the *m*_agg_ values for CPh6-PEtOx and CPh6-PiPrOx may be caused by the different hydrophobicity of their arms. Indeed, as shown by Winnik et al., PEtOx dehydration begins at about 50 °C, while in the case of PiPrOx, this can occur already at 20 °C [7]. Consequently, in CPh6-PiPrOx molecules at 21 °C, there can be a noticeable number of dehydrated units, the intermolecular interactions of which promote aggregation and, in particular, an increase in the size of micelle-like structures.

In addition to unimolecular micelles or small aggregates, large aggregates with a hydrodynamic radius *R*_s_ from 70 to 110 nm were detected in CPh6-PAlOz and CPh6-PAlOx solutions. No systematic change in *R*_s_ with concentration was found. The average values of the hydrodynamic radii <*R*_s_> of these supramolecular structures for the studied polymers are practically the same (Table 1). It can be assumed that these aggregates were formed mainly by “defective” molecules, the number of arms which was less than six, and the arm length was less than the average for the sample. The relative weight concentration *c*_s_ of large aggregates is low. The *c*_s_ value can be roughly estimated using the models of sphere for macromolecules and micelle-like structures and coil for large aggregates. As is known, the intensity *I*_i_ of *i*th specie is proportional to both the molar mass *M*_i_ and concentration *c* of particles [49,50,51]

*I*_i_ ~ *c*_i_*M*_i_ (2)

where *I*_i_ = *S*_i_*I*, *I* is integral light scattering intensity of solution, and *S*_i_ is relative contribution of *i*th specie in the *I* value. *S*_i_ = *S*_f_ and *c*_i_ = *c*_f_ for macromolecules, *S*_i_ = *S*_m_ and *c*_i_ = *c*_m_ for micelle-like structures, and *S*_i_ = *S*_s_ for large supramolecular structures (see Figure 3). The particle radius *R*_i_ is related to its molar mass as *M*_i_ ~ *R*_i_*^x^*. Parameter *x* depends on the particle shape, for example, *x* = 3 for spherical particles, *x* = 2 for coil structures, and *x* = 1 for rigid rods. Within the described approximations, we obtain for CPh6-PAlOz

*c*_s_/*c*_f_ = (*S*_f_/*S*_s_*) (R*_s_^2^/*R*_f_^3^) (3)

and CPh6-PAlOx

*c*_m_/*c*_f_ = (*S*_m_/*S*_s_*) (R*_s_^2^/*R*_m_^3^) (4)

The contributions *S*_i_ of different particles for solutions of investigated stars did not depend on polymer concentration. The average values of *S*_f_, *S*_m_, and *S*_s_ (<*S*_f_>, <*S*_m_>, and <*S*_s_>, respectively) are shown in Table 1. Substitution of <*S*_f_>, <*S*_m_>, and <*S*_s_> into Equations (3) and (4) gives values of relative concentration *c*_s_ of large aggregates for each polymer studied (Table 1). It is clearly seen that *c*_s_ did not exceed 8%.

In order to estimate the aggregation degree *m*_agg_ for large aggregates, one can use the previously proposed approach [52] based on comparing the translational friction coefficients for macromolecules and aggregates. By modeling unimolecular micelles by spheres, and large aggregates by ellipsoids of revolution, it is possible to show that the aggregation degree associated with the parameters of the ellipsoid by the equation [52]:

*m*_agg_ = *V*_ell_/*V*_sph_ = *p*^2^*R*_f_^3^/8*a*^3^ (5)

where *V*_ell_ is the volume of the modeling ellipsoid of revolution, *V*_sph_ is the volume of the modeling sphere, *p* = *a*/*b*, *a* and *b* are the major and minor axes of ellipsoid. Herein, the dependences of *a* and *b* on *p* for a model ellipsoid of revolution with a translational friction coefficient *f* are described by the formulas

*a* = *f*/(6πη_0_*F*(*p*)) (6)

*b* = *f*/(6πη_0_*pF*(*p*)) (7)

where

*aF*(*p*) = (*p*^2^−1)^1/2^/(*p* ln((*p* + (*p*^2^−1)^1/2^)/(*p*−(*p*^2^−1)^1/2^)) (8)

For a given value of the translational friction coefficient of the ellipsoid, the *m*_agg_ value turns out to be a rather weak function of the ellipsoid parameters. In the region of “reasonable” values of the asymmetry factor 1 < *p* < 3, the change in *m*_agg_ is about 30%. In particular, taking into account the values of *R*_h-D_ and *R*_s_ from Table 1, at *p* = 2, the values of *m*_agg_ are from 10,000 to 20,000 for CPh6-PEtOz and CPh6-PiPrOz and from 15,000 to 25,000 for CPh6-PEtOx and CPh6-PiPrOx. Therefore, for all investigated stars at low temperatures, the large aggregates contain from one to two tens of thousands of macromolecules.

Note that when estimating the relative weight concentration *c*_s_ of large aggregates and aggregation degree *m*_agg_, we used the hydrodynamic radii of the particles and their contributions to the integral value of light scattering, which were determined using a PhotoCor Complex setup. Similar information for CPh6-PEtOz and CPh6-PEtOx solutions was obtained using a Zetasizer Nano ZS particle analyzer. Figure S1 in Supplementary Materials shows the corresponding distributions for solutions of these polymers. As might be expected, they are qualitatively similar to the dependencies shown in Figure 3. The average values of hydrodynamic radii *R*_f_, *R*_m_ and *R*_s_ obtained with different instruments coincide within the experimental error. A similar situation takes place for the contributions of particles to the integral light scattering intensity. However, one must keep in mind that the accuracy of determining these parameters is significantly lower, and the experimental error can reach 15%.

### 3.2. Behavior of Star-Shaped Six-Arm Pseudo-Polypeptoids in Aqueous Solutions on Heating

**Figure 5:**
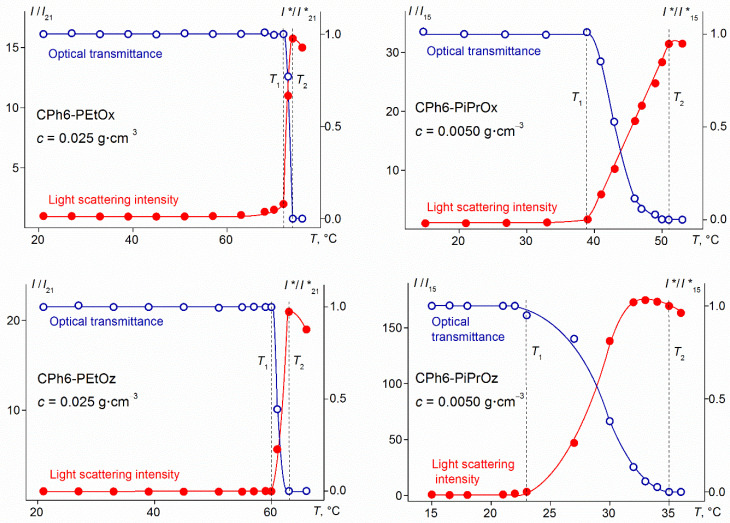
Temperature dependences of relative light scattering intensity *I*/*I*_15_ and *I*/*I*_21_ and relative transmission I*/I*_15_ and I*/I*_21_ for solutions of investigated star polymers. *I*_15_ and *I*_21_ are light scattering intensity at 15 °C and 21 °C, respectively. I*_15_ and I*_21_ are optical transmission at 15 °C and 21 °C, respectively.

**Figure 6:**
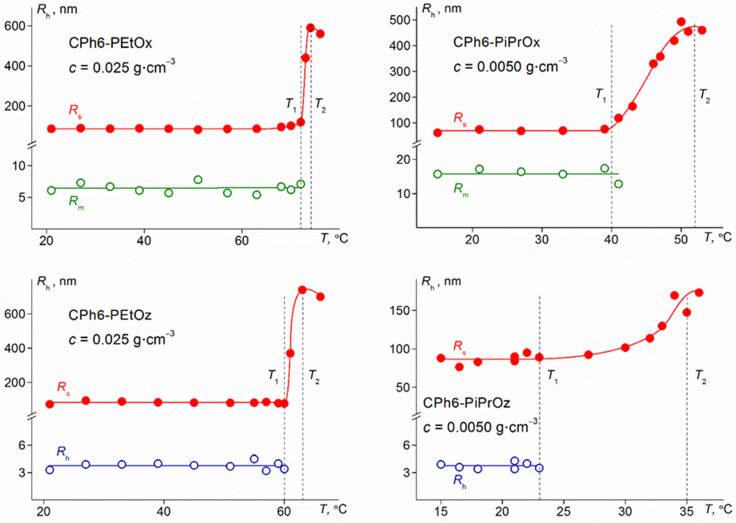
Temperature dependences of hydrodynamic radii *R*_h_ of scattering objects for aqueous solutions of star-shaped pseudo-polypeptoids.

A phase transition was detected in aqueous solutions of the studied polymers on heating by methods of light scattering, turbidimetry, and microcalorimetry. The temperature *T*_1_ of the onset of phase separation was determined from the beginning of a sharp decrease in optical transmission I* and the beginning of a strong increase in the light scattering intensity *I* (Figure 5). Below *T*_1_, the characteristics of most of the studied solutions did not change with temperature (Figure 5 and Figure 6). Only in some solutions with PAlOx arms, when approaching *T*_1_, a slight increase in intensity *I* was observed, caused by an increase in the size *R*_s_ of large aggregates and their contribution *S*_s_ to the total intensity of light scattering. This behavior distinguishes the studied polymers from the star-shaped PAlOz and PAlOx, whose cores were calix [*n*] arenes (*n* = 4, 8) [46,53]. In solutions of the mentioned polymers, the processes of aggregation and self-organization at the molecular level began long before reaching *T*_1_. Probably, this is the manifestation of the role of the branching center in the formation of the properties of thermosensitive stars in aqueous solutions.

Above *T*_1_, the light scattering intensity increased very rapidly with temperature, achieving the maximum value near the temperature *T*_2_, at which the optical transmission became zero (Figure 5). *T*_2_ can be considered as the temperature of the finishing of the phase separation according to turbidimetry data. The reason for the observed behavior of light scattering is the growth of radius of the large aggregates (Figure 6) which is often observed for thermosensitive polymer stars [43,46,54,55,56]. At *T* ≥ *T*_1_, macromolecules in solutions of CPh6-PAlOz and small aggregates in solution of CPh6-PAlOx were not detected by the dynamic light scattering. Thus, at *T* > *T*_1_, the dominant process in solutions of the studied star-shaped polymers was aggregation as a result of an increase in dehydration degree on heating and the formation of intermolecular hydrogen bonds. Above *T*_2_, a decrease in the *I* and *R*_s_ values was observed, however, a quantitative analysis of these data is impossible, since in this region the solutions are turbid and light scattering is multiple.

**Figure 7:**
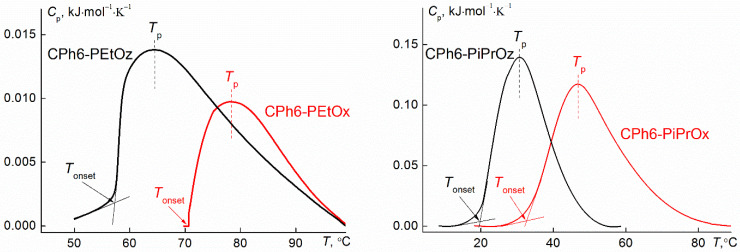
Microcalorimetric endotherms of CPh6-PiPrOz and CPh6-PiPrOx (*c* = 0.015 g cm^−3^) and CPh6-PEtOz and CPh6-PEtOx (*c* = 0.025 g cm^−3^) in water (rate 1.0 °C/min).

**Figure 8:**
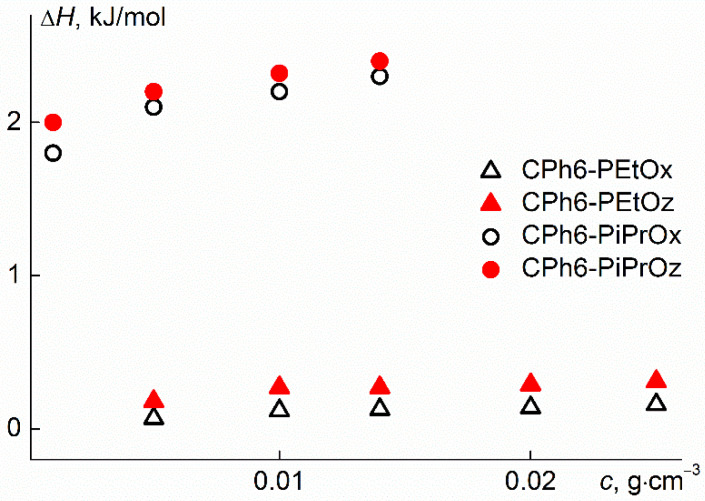
Concentration dependences of enthalpy for the studied aqueous solutions.

The microcalorimetric endotherms of the aqueous solutions of CPh6-PAlOx and CPh6-PAlOz are shown in Figure 7. The dependences of the phase transition heat on the concentration Δ*H* for the studied polymer stars had a form typical for thermosensitive polymers (Figure 8). Thus, no qualitative change in thermodynamic behavior was observed on passage from linear polymers to star-shaped polymers. On the other hand, the influence of the arm structure was clearly visible. As can be seen in Figure 8, for polymer stars containing isopropyl groups, the Δ*H* value was almost an order of magnitude higher than the phase transition heat for CPh6-PEtOz and CPh6-PEtOx, that can explain the lower hydrophobicity of the latter. It should also be noted that Δ*H* was slightly higher for the star-shaped CPh6-PAlOz in comparison with this characteristic for CPh6-PAlOx.

From the obtained values of Δ*H*, the temperatures of the peak maximum and the maximum heat capacities, one can calculate the Van’t Hoff enthalpy Δ*H*^vH^ [57,58]:

Δ*H*^vH^ = 4*RT*_p_^2^(C_p_^max^/Δ*H*) (9)

where *T*_p_ is the temperature of the maximum, *C*_p_^max^ is the heat capacity at *T*_p_, and *R* is universal gas constant. The ratios *n*’ = Δ*H*^vH^/Δ*H* gives information about the number of structural units of the polymers that cooperate with each other in the transition [59], i.e., so-called “cooperative units”. The values of Δ*H*^vH^, Δ*H* and *n*’ given in Table 2 were obtained for solutions with the maximum polymer concentration: *c* = 0.015 g cm^−3^ for CPh6-PiPrOz and CPh6-PiPrOx and *c* = 0.025 g cm^−3^ for CPh6-PEtOz, CPh6-PEtOx. The highest *n*’ value was obtained for CPh6-PEtOx, which is the most hydrophilic, while for the most hydrophobic CPh6-PiPrOz, the *n*’ parameter has the lowest value.

### 3.3. Concentration Dependences of Phase Separation Temperatures for Aqueous Solutions of Investigated Polymer Stars

**Figure 9:**
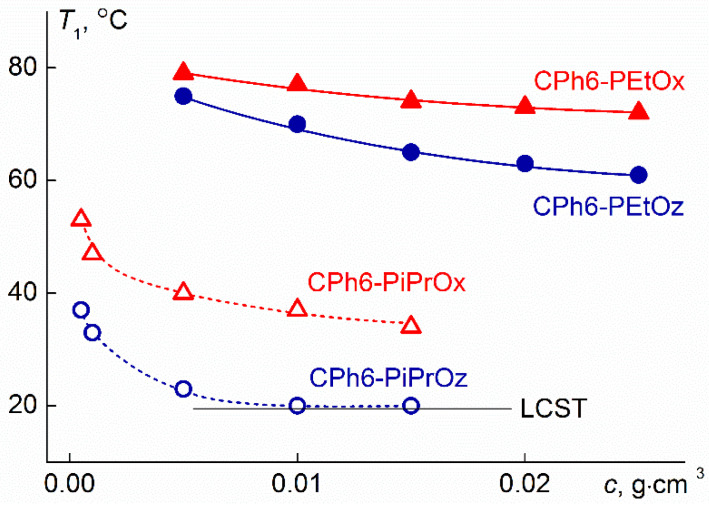
Concentration dependencies of the phase separation temperature *T*_1_ for star-shaped CPh6-PAlOz and CPh6-PAlOx.

Figure 9 shows the concentration dependences of temperature *T*_1_ obtained for the studied polymers. The phase separation temperatures increased with dilution. This is typical for dilute solutions of thermosensitive polymers. Table 3 compares the phase separation temperatures according to turbidimetry (*T*_1_ and *T*_2_) and microcalorimetry (*T*_onset_ and *T*_p_) data (*T*_onset_ is temperature of the onset of phase separation (see Figure 7)). For all polymers, the temperatures of the onset of phase separation, determined by discussed methods, differed insignificantly. On the other hand, the values of *T*_2_ and *T*_p_ for stars with arms containing isopropyl groups differed by 8 °C. The observed distinctions can be associated with both different physical bases of the methods and with different experimental procedures (discrete temperature variation and constant heating).

The lower critical solution temperature (LCST) was determined only for CPh6-PiPrOz. Taking into account the character of the dependences of *T*_1_ on *c*, it can be assumed that for other samples LCST slightly differs from the *T*_1_ value obtained at the maximum studied concentration. As for the absolute values of the phase transition temperatures, for stars with oxazoline arms, they do not differ very much from the cloud points for linear [6,20,33,60] and star-shaped [26,27,33,53,61] PEtOx and PiPrOx. For PAlOz, the data are much less even for linear polymers [20,24], and for star-shaped CPh6-PAlOz they are absent at all. Besides, when analyzing the phase separation temperatures, it is necessary to take into account the influence of at least the molar mass of the polymer and the hydrophilic-hydrophobic balance [33], and it is rather difficult to draw reliable conclusions about the “chemical structure—macromolecule architecture—phase separation temperature” correlations.

As expected, for stars with more hydrophilic arms CPh6-PEtOz and CPh6-PEtOx, higher phase separation temperatures were obtained in comparison with *T*_1_ for the CPh6-PiPrOz and CPh6-PiPrOx solutions. This difference is quite large, at a given concentration it is 40–45 °C and correlates with the data for linear PEtOx and PiPrOx [20,33,60]. At passage from PAlOx to PAlOz, the hydrophobicity of the polymer increases as a result of the elongation of the monomer unit by one –CH_2_– group. This leads to a decrease in the phase separation temperature. In the investigated concentration range, in the pair of CPh6-PEtOz and CPh6-PEtOx, the *T*_1_ values differ by 5–10 °C, and for CPh6-PiPrOz and CPh6-PiPrOx the distinction is more (from 14 to 17 °C). It should be borne in mind that CPh6-PEtOz and CPh6-PiPrOz are characterized by higher molar mass. This should have led to a decrease in the phase separation temperatures for stars with PAlOz arms. Therefore, the discussed difference in the* T*_1_ values for CPh6-PAlOz and CPh6-PAlOx could be slightly smaller if the compared samples had similar molar masses and hydrophilic-hydrophobic balance.

Note that the values of the phase separation temperatures and LCST can be significantly influenced by the polydispersion of the samples [62]. Therefore, the dispersion should be taken into account when analyzing the *T*_1_ values. However, as can be seen from Table 1, the values of *Đ* for the studied CPh6-PAlOz and CPh6-PAlOx are close. Accordingly, it can be assumed that the effect of polydispersion will be minimal.

The effect of the structure of the studied stars is most pronounced when comparing CPh6-PEtOz and CPh6-PiPrOx. The monomer units of the arms of these polymers have the same set of atoms and groups, but for CPh6-PEtOz the phase separation temperatures are about 40 °C higher than *T*_1_ for CPh6-PiPrOx. Consequently, the position of the –CH_2_– group, namely in the main chain or in the side fragments, is a decisive factor determining the phase separation temperature. Note also that the difference in the *T*_1_ values for CPh6-PEtOz and CPh6-PEtOx is significantly less than the corresponding difference for CPh6-PiPrOz and CPh6-PiPrOx, i.e., the higher the hydrophobicity of the homologues, the greater the difference in the phase separation temperatures of their solutions.

## 4. Conclusions

Aqueous solutions of star-shaped six-arm pseudo-polypeptoids on heating were investigated within a wide concentration and temperature ranges. Poly-2-ethyl-2-oxazine, poly-2-isopropyl-2-oxazine, poly-2-ethyl-2-oxazoline, and poly-2-isopropyl-2-oxazoline were arms and hexaaza [2_6_] orthoparacyclophane was core.

At low temperatures, the behavior of the solutions depended on the structure and size of arms. Macromolecules, more precisely unimolecular micelles, and large aggregates were observed in solutions of poly-2-alkyl-2-oxazines, while in solutions of poly-2-alkyl-2-oxazolines there were two types of aggregates. The formation of the smaller ones is caused by interaction of hydrophobic CPh6 core, because the short arms do not sufficiently shield them from the solvent. In addition to unimolecular micelles and small aggregates, in aqueous solutions of the studied polymer stars, large loose aggregates were present, which contained from 10,000 to 25,000 macromolecules. These supramolecular structures were formed mainly from molecules, the arm number in which was less than six, and the arm length was less than the average for the sample. The weight fraction of large aggregates did not exceed 8%.

On heating below the phase separation temperature, in contrast to the previously studied pseudo-polypeptoids with a calix[*n*]arene core, the characteristics of the CPh6-PAlOz and CPh6-PAlOx solutions were practically independent of temperature. This behavior can be explained by the influence of the structure of the branching center. Within the phase separation interval, the prevailing process was aggregation due to the formation of intermolecular hydrogen bonds. This led to the fact that unimolecular micelles and small aggregates attached to large aggregates or formed new supramolecular structures, the size of which increased on heating.

For all investigated solutions, the phase separation temperatures increased with dilution, but LCST was determined reliably only for star with poly-2-isopropyl-2-oxazine arms. At a given concentration, the phase separation temperatures for poly-2-alkyl-2-oxazoline stars with CPh6 core differed from their values for linear analogs and stars with branch centers of a another structure. These differences are insignificant and can be caused both by the difference in the structure and architecture of the molecules of the compared samples, and by the influence of the molar mass of the polymers. It was shown that an increase in the arm hydrophobicity leads to a decrease in the phase separation temperature of the aqueous solutions of studied stars. In particular, their values for poly-2-alkyl-2-oxazolines are higher than for poly-2-alkyl-2-oxazines. On passage from CPh6-PEtOz to CPh6-PiPrOz and from CPh6-PEtOx to CPh6-PiPrOx, the phase separation temperatures decreased. Besides, for polymer stars containing isopropyl groups, the phase transition heat was almost an order of magnitude higher than the Δ*H* value for star with ethyl groups in side fragment of arms.

## Figures and Tables

**Table 1:** Molar masses and average values of hydrodynamic radii of scattering object in aqueous solutions of investigated stars-shaped CPh6-PAlOx and CPh6-PAlOz.

Polymer	*M*_sD_, ^(1)^ g mol^−1^	*Đ* ^(2)^	*R*_h-D_, ^(1)^ nm	<*R*_f_>, nm	<*R*_m_>, nm	<*R*_s_>, nm	<*S*_f_>	<*S*_m_>	<*S*_s_>	*C*_s_
CPh6-PEtOz	23,000	1.27	3.5	3.9 ± 0.3	-	89 ± 8	0.09	-	0.91	0.07
CPh6-PiPrOz	20,000	1.24	3.3	4.1 ± 0.3	-	89 ± 9	0.10	-	0.92	0.07
CPh6-PEtOx	15,000	1.29	3.0	-	6.3 ± 0.7	84 ± 7	-	0.35	0.65	0.06
CPh6-PiPrOx	14,000	1.19	2.6	-	15 ± 1	86 ± 12	-	0.84	0.16	0.08

^(1)^ the values of *M*_sD_ and *R*_h-D_ were obtained in [25]. ^(2)^ the values of *Đ* were calculated using GPC curves which were obtained in [25].

**Table 2:** Thermodynamic characteristics of solutions of star-shaped pseudo-polypeptoids.

Polymer	Δ*H*^vH^, kJ/mol	Δ*H*, kJ/mol	*n*’
CPh6-PEtOz	6.0	0.3	20
CPh6-PiPrOz	1.8	2.4	0.75
CPh6-PEtOx	10.1	0.2	50
CPh6-PiPrOx	3.4	2.2	1.5

**Table 3:** Phase separation temperatures of solutions of star-shaped pseudo-polypeptoids at concentration *c* = 0.015 g cm^−3^.

Sample	*T*_onset_, °C	*T*_p_, °C	*T*_1_, °C	*T*_2_, °C
CPh6-PEtOz	63	70	65	70
CPh6-PiPrOz	20	31	20	23
CPh6-PEtOx	75	83	74	-
CPh6-PiPrOx	33	47	34	39

## Data Availability

Not applicable.

## Supplementary Materials

The following are available online at https://www.mdpi.com/article/10.3390/polym13091429/s1, Figure S1: The distributions of scattering species over hydrodynamic radii for solutions of star-shaped CPh6-PEtOx and CPh6-PEtOz. Rf, Rm, and Rs, are the hydrodynamic radii of macromolecules (fast mode), small aggregates (middle mode) and large aggregates (slow mode), respectively.
