# Supplementary material for: Self-Organization in Dilute Aqueous Solutions of Thermoresponsive Star-Shaped Six-Arm Poly-2-Alkyl-2-Oxazines and Poly-2-Alkyl-2-Oxazolines
Source: Polymers (Basel). 2021 Apr 29;13(9):1429. doi: 10.3390/polym13091429 (PMC8125547; doi:10.3390/polym13091429)
Supplement: Supplementary file 1 [file polymers-13-01429-s001.zip › polymers-1184046-supplementary.pdf]

# Supplementary Materials

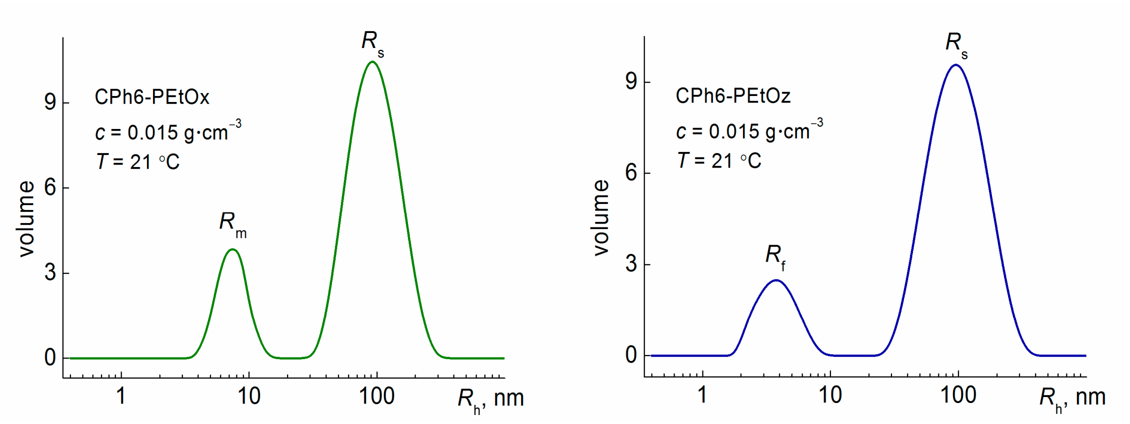


**Figure S1**. The distributions of scattering species over hydrodynamic radii for solutions of star-shaped CPh6-PEtOx and CPh6-PEtOz. Rf, Rm, and Rs, are the hydrodynamic radii of macromolecules (fast mode), small aggregates (middle mode) and large aggregates (slow mode), respectively
